# Modeling human enteric dysbiosis and rotavirus immunity in gnotobiotic pigs

**DOI:** 10.1186/s13099-016-0136-y

**Published:** 2016-11-08

**Authors:** Erica L. Twitchell, Christine Tin, Ke Wen, Husen Zhang, Sylvia Becker-Dreps, M. Andrea Azcarate-Peril, Samuel Vilchez, Guohua Li, Ashwin Ramesh, Mariah Weiss, Shaohua Lei, Tammy Bui, Xingdong Yang, Stacey Schultz-Cherry, Lijuan Yuan

**Affiliations:** 1 Department of Biomedical Sciences and Pathobiology, Virginia-Maryland College of Veterinary Medicine, Virginia Polytechnic Institute and State University, Blacksburg, VA USA; 2 Microbiome Core, Cancer Inflammation Program, National Cancer Institute, Bethesda, MD USA; 3 Department of Family Medicine, University of North Carolina School of Medicine, Chapel Hill, NC USA; 4 Department of Cell Biology and Physiology, School of Medicine and Microbiome Core Facility, Center for Gastrointestinal Biology and Disease, University of North Carolina, Chapel Hill, NC USA; 5 Department of Microbiology and Parasitology, Faculty of Medical Sciences, National Autonomous University of Nicaragua, León, Nicaragua; 6 Department of Infectious Diseases, St. Jude Children’s Research Hospital, Memphis, TN USA

**Keywords:** Enteric dysbiosis, Gnotobiotic pig, Rotavirus, Vaccine, Enteric immunity

## Abstract

**Background:**

Rotavirus vaccines have poor efficacy in infants from low- and middle-income countries. Gut microbiota is thought to influence the immune response to oral vaccines. Thus, we developed a gnotobiotic (Gn) pig model of enteric dysbiosis to study the effects of human gut microbiota (HGM) on immune responses to rotavirus vaccination, and the effects of rotavirus challenge on the HGM by colonizing Gn pigs with healthy HGM (HHGM) or unhealthy HGM (UHGM). The UHGM was from a Nicaraguan infant with a high enteropathy score (ES) and no seroconversion following administration of oral rotavirus vaccine, while the converse was characteristic of the HHGM. Pigs were vaccinated, a subset was challenged, and immune responses and gut microbiota were evaluated.

**Results:**

Significantly more rotavirus-specific IFN-γ producing T cells were in the ileum, spleen, and blood of HHGM than those in UHGM pigs after three vaccine doses, suggesting HHGM induces stronger cell-mediated immunity than UHGM. There were significant correlations between multiple Operational Taxonomic Units (OTUs) and frequencies of IFN-γ producing T cells at the time of challenge. There were significant positive correlations between *Collinsella* and CD8+ T cells in blood and ileum, as well as CD4+ T cells in blood, whereas significant negative correlations between *Clostridium* and *Anaerococcus*, and ileal CD8+ and CD4+ T cells. Differences in alpha diversity and relative abundances of OTUs were detected between the groups both before and after rotavirus challenge.

**Conclusion:**

Alterations in microbiome diversity and composition along with correlations between certain microbial taxa and T cell responses warrant further investigation into the role of the gut microbiota and certain microbial species on enteric immunity. Our results support the use of HGM transplanted Gn pigs as a model of human dysbiosis during enteric infection, and oral vaccine responses.

**Electronic supplementary material:**

The online version of this article (doi:10.1186/s13099-016-0136-y) contains supplementary material, which is available to authorized users.

## Background

Rotavirus gastroenteritis accounted for approximately 37% of all diarrhea-related deaths and 5% of all deaths in children less than 5 years of age; in all, rotavirus killed approximately 453,000 children in 2008, mostly from low-middle income countries (LMICs) [1, 2]. Oral vaccines for rotavirus, poliovirus, cholera and shigellosis are less efficacious in children from LMICs than in children from higher income countries [3, 4]. Specifically regarding oral rotavirus vaccination, the two commercially available vaccines (RotaTeq® and Rotarix®) only have 39–70% efficacy, with an average of 50–60% efficacy in LMICs; whereas rotavirus vaccines are 80–90% effective in high-income countries [3, 5–7]. Differences in rotavirus vaccine efficacy may be due to a combination of factors including environmental enteric dysfunction (EED), variations in the gut microbiome, an altered gut microbiota composition (dysbiosis), high maternal antibody titers transferred through the placenta or breast milk, malnutrition, or influence of concurrent enteropathogens [3, 4, 6–8].

Environmental enteric dysfunction, also known as environmental enteropathy is a structural and functional disorder of the small intestine that is most frequently seen in children from low income settings [9]. EED leads to gut barrier disruption, nutrient malabsorption, impaired gut immune function, and ultimately, oral vaccine failure, growth stunting, and delayed cognitive development [9–11]. However, children with EED do not always have diarrhea [10]. Histologic features include villous blunting, crypt hyperplasia, and lymphocytic infiltration of the epithelium and lamina propria in the small intestine [9]. The cause of this condition is unknown, but theories include constant exposure to enteropathogens in food, water, or the environment, imbalance of gut microbiota, and an altered immune response triggered by intestinal microbes [4, 9, 10].

The gastrointestinal microbiota is important for the development of enteric immunity, prevention of enteropathogen colonization, and utilization of dietary energy [4, 12]. The composition of the gut microbiota in children is influenced by the method of delivery (vaginal or C-section), environmental hygiene, and nutritional status [4]. Studies have shown that the composition of gut microbiota is significantly different between African and northern European infants, and between malnourished and well-nourished children [10, 12, 13].

The goal of this study was to create a gnotobiotic (Gn) pig model of human enteric dysbiosis to evaluate the influence of the gut microbiota on immune responses to an attenuated human rotavirus vaccine (AttHRV). Since pigs and humans share high genomic and protein sequence homologies, an omnivorous diet, colonic fermentation, and similar immune systems, pigs serve as valuable models in biomedical research [14]. To this effect, the neonatal Gn pig model is a proven model of human rotavirus disease and immunity [15]. Protection against rotavirus diarrhea in the Gn pig model is positively correlated with frequencies of intestinal IFN-γ producing T cells, rotavirus-specific serum IgA, intestinal IgA and intestinal IgG antibody titers, and IgA antibody secreting cells in the intestine [16–18].

The study of microbiota on virus life cycle is facilitated by the germ-free condition in Gn pigs [19–22]. In addition, our previous studies of human gut microbiota (HGM) transplanted Gn pigs demonstrated that HGM from a healthy C-section delivered infant was able to colonize newborn Gn pig intestine and similar microbiota was observed between HGM from the infant and colonized Gn pigs [20, 21]. In this study we attempted to develop a Gn pig model of human enteric dysbiosis by colonizing Gn pigs with unhealthy HGM (UHGM). UHGM designates the HGM samples from children with evidence of gut inflammation and permeability (based on enteropathy scores (ES) and an impaired immune response to the rotavirus vaccine RV5 (RotaTeq®), suggesting an unhealthy gut. HGM from children with low ES and a robust immune response to RV5, suggesting a normal gut, was designated as healthy HGM (HHGM). We hypothesized that HHGM in Gn pigs would induce a stronger immune response after vaccination than the dysbiosis pigs, and that certain components of the HGM may be correlated with impaired immune responses.

## Results

### Antibody response in HHGM and UHGM pigs

**Fig. 1:**
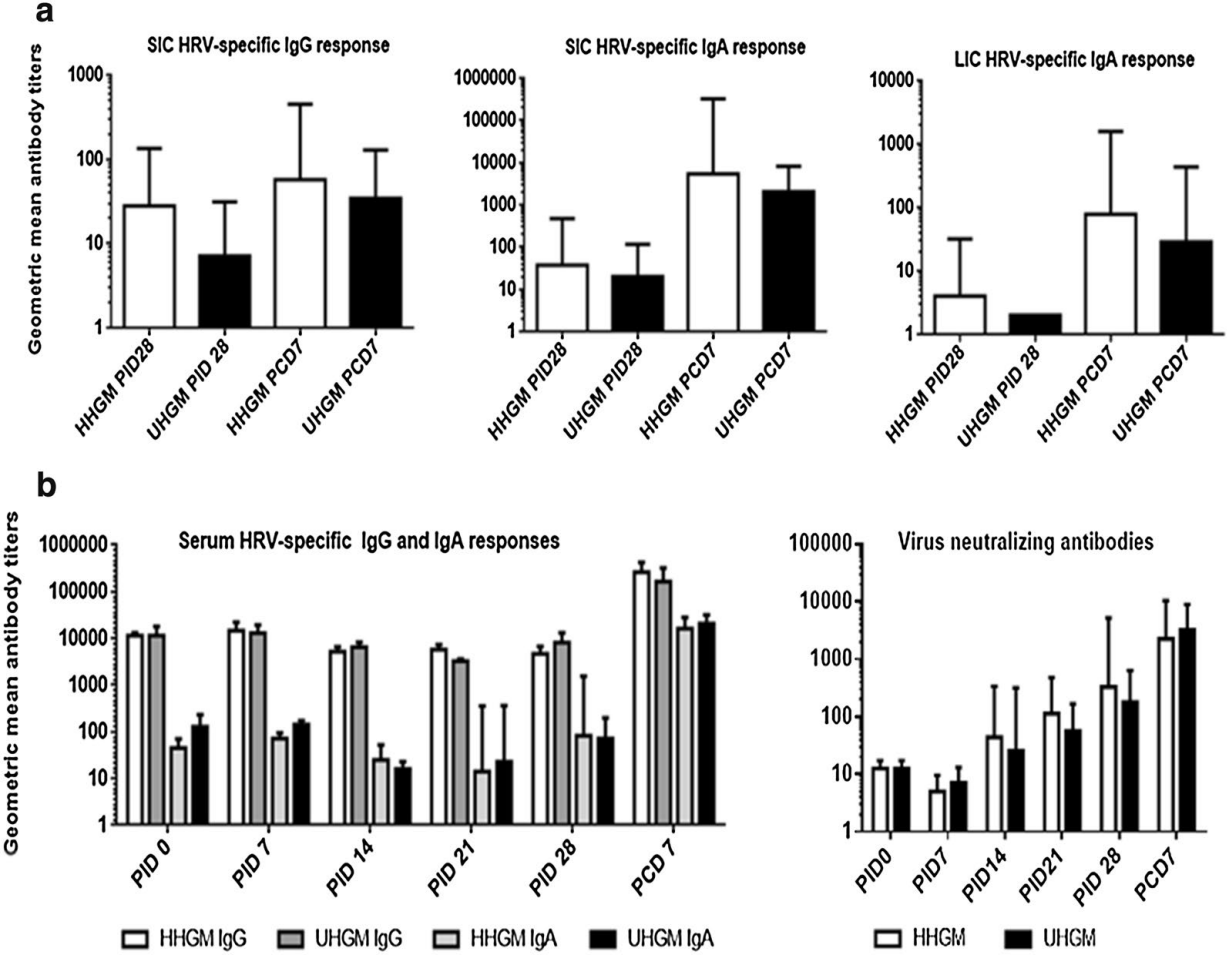
Rotavirus-specific antibody responses. Rotavirus-specific IgG and IgA antibody responses in small and large intestinal contents (**a**) and IgG, IgA and virus neutralizing antibody response in serum (**b**). *Error bars* are represented as standard error of mean. Kruskal–Wallis rank sum test was used for comparisons. There are no significant differences between the groups.* SIC* small intestinal contents;* LIC* large intestinal contents;* PID* post-inoculation day;* PCD* post-challenge day

Healthy HGM and UHGM samples were orally inoculated into a subset of newborn Gn pigs, respectively. After AttHRV inoculation and virulent HRV (VirHRV) challenge, antibody titers were measured in small intestinal contents (SIC), large intestinal contents (LIC), and serum. Rotavirus-specific immunoglobulin titers in intestinal contents from HHGM pigs consistently trended higher than titers from the UHGM pigs, before and after VirHRV challenge, including IgG and IgA in SIC and IgA in LIC (Fig. 1a). However, rotavirus-specific IgA, IgG, and virus neutralizing antibody responses in serum did not differ between the two groups at any time point (Fig. 1b). These data suggest that HHGM could have an influence toward a stronger mucosal immune response to oral rotavirus vaccine than UHGM.

### Virus specific effector T cell response

**Fig. 2:**
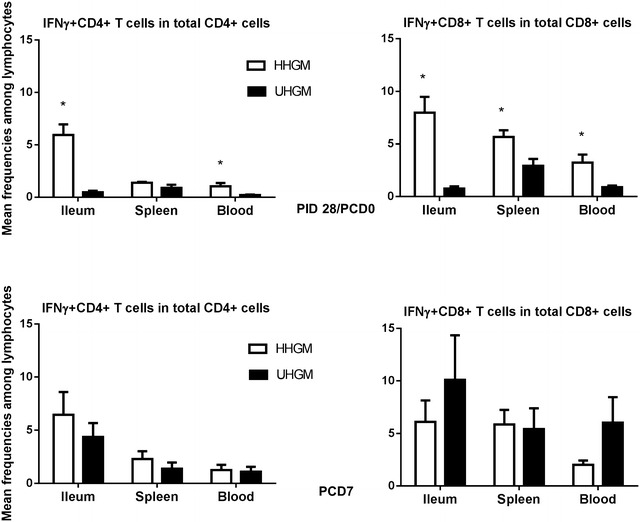
Frequencies of IFN-γ producing CD8+ and CD4+ T cells. Frequencies of IFN-γ producing CD8+ and CD4+ T cells among total CD3+CD8+ and CD3+CD4+ cells on PID28/PCD0 (*upper panel*) and PCD7 (*lower panel*) in ileum, spleen and blood of HHGM verses UHGM colonized pigs. *Error bars* indicate standard errors of the mean. *Asterisks* indicate significant differences when compared to PM25 pigs (Kruskal–Wallis rank sum test, *p* < 0.05; *n* = 5–7)

Frequencies of IFN-γ+CD8+ T cells among total CD8+ T cells in the ileum, spleen, and blood of HHGM pigs were significantly higher than those in UHGM pigs at the time of VirHRV challenge (Fig. 2). Frequencies of IFN-γ+CD4+ T cells among total CD4+ T cells in the ileum and blood were also significantly higher in HHGM pigs than in UHGM pigs at the time of VirHRV challenge. After challenge, IFN-γ+ CD8+ and IFN-γ+CD4+ T cell responses did not differ significantly between the two groups in any tissue. The data demonstrate that the AttHRV vaccine induced significantly stronger anti-viral effector T cell immune responses in pigs colonized with HHGM than those with UHGM. The significantly higher virus-specific effector T cell responses at the time of challenge were associated with increased protection against rotavirus shedding and clinical signs (Fig. 2; Table 1).

**Table 1:** Clinical signs and rotavirus fecal shedding in Gn pigs after VirHRV challenge

Group	N	Clinical signs				Fecal virus shedding (by CCIF and/or ELISA)			
		% with diarrhea^a,^*	Mean days to onset^b,^**	Mean duration days^e,^**	Mean cumulative score^c,^**	% shedding virus*	Mean days to onset^b,^**	Mean duration days^e,^**	Mean peak titer (FFU/ml)
HHGM	7	57.1	4.3 (0.23)^d^	1.3 (0.57)^d^	7.7 (0.77)^d^	42.85*	1.7 (0.2)^d^	1.6 (0.7)^d^	657.4
UHGM	6	83.3	2.3 (1.1)	2.0 (0.89)	11.2 (1.2)	100*	2.4 (0.3)	3.0 (0.63)	1683.7

*ELISA* enzyme-linked immunosorbent assay, *CCIF* cell culture immunofluorescent assay, *FFU* fluorescence forming unit

* Fisher’s exact test was used for comparisons. Asterisk indicate significant differences among groups (n = 6–7; p < 0.05)

** Kruskal–Wallis rank sum test was used for comparisons. No statistically significant differences were observed between the groups

^a^ Pigs with daily fecal scores of ≥2 were considered diarrheic. Fecal consistency was scored as follows: 0, normal; 1, pasty; 2, semiliquid; and 3, liquid

^b^ In the groups where some but not all pigs had diarrhea or shedding, the onset of diarrhea or shedding for non-diarrheic/shedding pigs was designated as 8 for calculating the mean days to onset

^c^ Mean cumulative score calculation included all the pigs in each groups

^d^ Standard error of the mean

^e^ For days of diarrhea and virus shedding, if no diarrhea or virus shedding until the euthanasia day (PCD7), the duration days were recorded as 0

### Clinical signs and virus shedding

After challenge with VirHRV, HHGM pigs had significantly reduced incidence and shorter duration of viral shedding, and lower mean peak virus titer than UHGM pigs (Table 1). HHGM pigs had a slightly lower incidence, delayed onset, shorter duration of diarrhea, and lower cumulative diarrhea score compared to the UHGM pigs. These results suggest that HHGM is associated with less severe clinical signs and viral shedding than UHGM in vaccinated pigs subsequently challenged with VirHRV, indicating that HHGM facilitates the development of a stronger protective immunity.

### Microbiome analysis

**Fig. 3:**
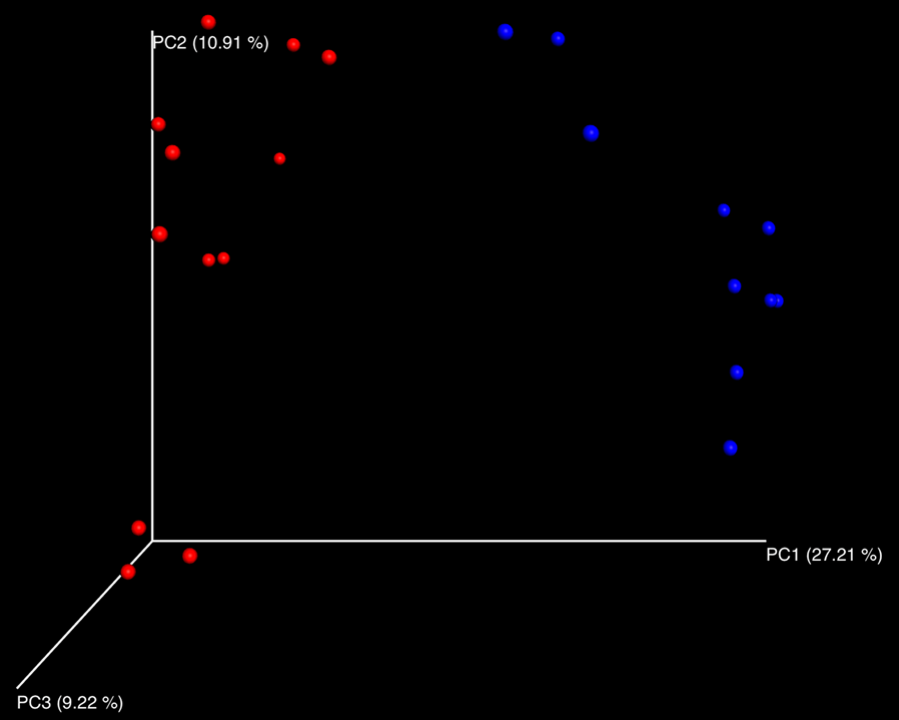
PCoA plot of the microbial communities in the large intestinal contents of Gn pigs. Communities were plotted based on unweighted UniFrac. *Blue dots* represent HHGM and red dots represent UHGM

Alpha diversity, measured by Shannon index, phylogenic diversity, observed species, and Chao 1 were compared between HHGM and UHGM pig groups (Table 2). Measurements of alpha diversity in HHGM pigs were significantly lower than those in UHGM pigs at post-inoculation day (PID) 28 and post-challenge day (PCD) 7. In addition, alpha diversity measurements decreased in HHGM pigs from PID28 to PCD7. There were no significant differences before or after challenge for the UHGM pigs. These results suggest that VirHRV challenge caused a greater disruption to the microbiota in HHGM pigs than in UHGM pigs. Beta diversity analysis was visualized with a PCoA plot based on unweighted UniFrac. Regardless of the time point, the microbiota from pigs with HHGM clustered in one group while samples from UHGM pigs formed another group (Fig. 3).

**Table 2:** Mean alpha diversity parameters in gut microbiome of HGM colonized Gn pigs

HHGM	Comparison between time points				Comparison between groups		
	PID28	PCD7	p**	PID28	HHGM	UHGM	p
Shannon index	2.366	1.236	0.009	Shannon index	2.366	2.743	0.006
Phylogenetic diversity	6.806	6.108	0.016	Phylogenetic diversity	6.806	6.580	0.855
Observed species	92.460	65.180	0.009	Observed species	92.460	90.550	0.855
Chao 1	168.399	125.946	0.009	Chao 1	168.399	189.092	0.201

UHGM	Comparison between time points				Comparison between groups		
	PID28	PCD7	p	PCD7	HHGM	UHGM	p
Shannon index	2.743	2.780	0.749	Shannon index	1.236	2.780	0.006
Phylogenetic diversity	6.580	6.988	0.262	Phylogenetic diversity	6.108	6.988	0.045
Observed species	90.550	102.583	0.173	Observed species	65.180	102.583	0.006
Chao 1	189.092	206.175	0.631	Chao 1	125.946	206.175	0.006

** Kruskal–Wallis rank sum test was used for comparisons. p < 0.05 is considered significant

**Fig. 4:**
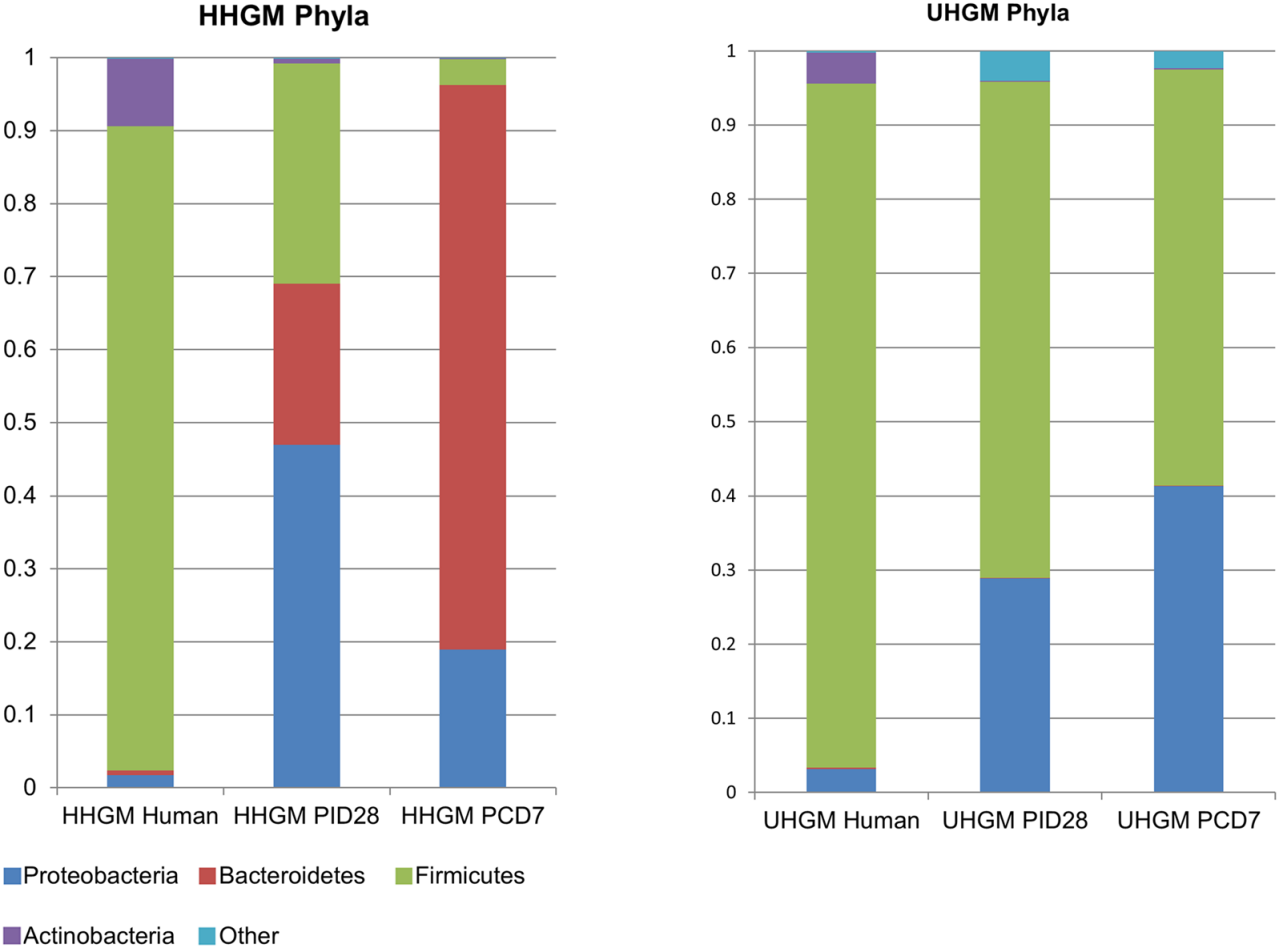
Mean relative abundance of phyla in the microbial community of large intestinal contents. Mean relative abundance of phyla in the microbial community of large intestinal contents of HGM-colonized Gn pigs before HRV challenge (PID28/PCD0) and postchallenge (PCD7) compared to infant HGM. *Numbers* represent the single stool sample from infant HGM and averages for the pig groups (n = 5–6)

**Fig. 5:**
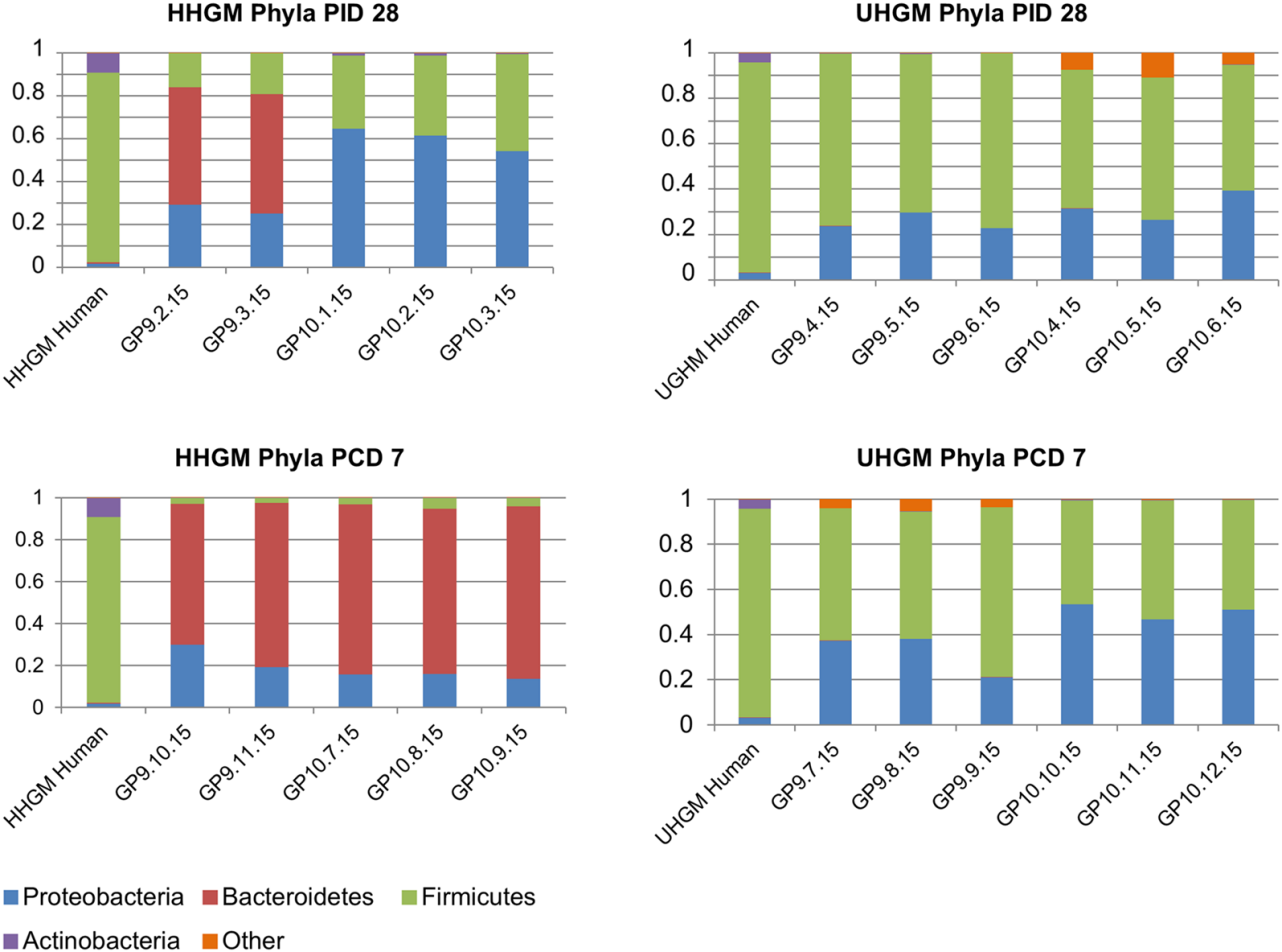
Relative abundance of phyla in the microbial community of large intestinal contents of individual pigs. Relative abundance of phyla in the microbial community of large intestinal contents of individual HGM-colonized Gn pigs before VirHRV challenge (PID28/PCD0) and postchallenge (PCD7). The HHGM human sample is included for reference in each panel. HHGM human is the sample from child SV14 and UHGM human is the sample from child PM25

In HGM transplanted pigs, phyla represented in UHGM pigs were similar to those in the human infant samples, with Firmicutes being the most abundant. Firmicutes was also the most abundant phylum in the healthy human infant stool sample. Conversely, Proteobacteria or Bacteroidetes was the most abundant phyla in HHGM pigs with Firmicutes being second or third in mean relative abundance (Fig. 4). There were significantly more Firmicutes in UHGM pigs than in HHGM pigs at PID28. After VirHRV challenge on PCD7, the phyla, Firmicutes, Proteobacteria and Tenericutes had significantly higher mean relative abundance in the UHGM pigs while mean relative abundance of Bacteroidetes was significantly higher in HHGM pigs. When evaluating microbiome shifts in HHGM pigs before and after VirHRV, Firmicutes, Proteobacteria and Verrucomicrobia were shown to be significantly decreased while Bacteroidetes significantly increased in mean relative abundance. There were no significant changes in gut phyla composition in UHGM between pre- and post-VirHRV challenge samples (Fig. 5).

**Fig. 6:**
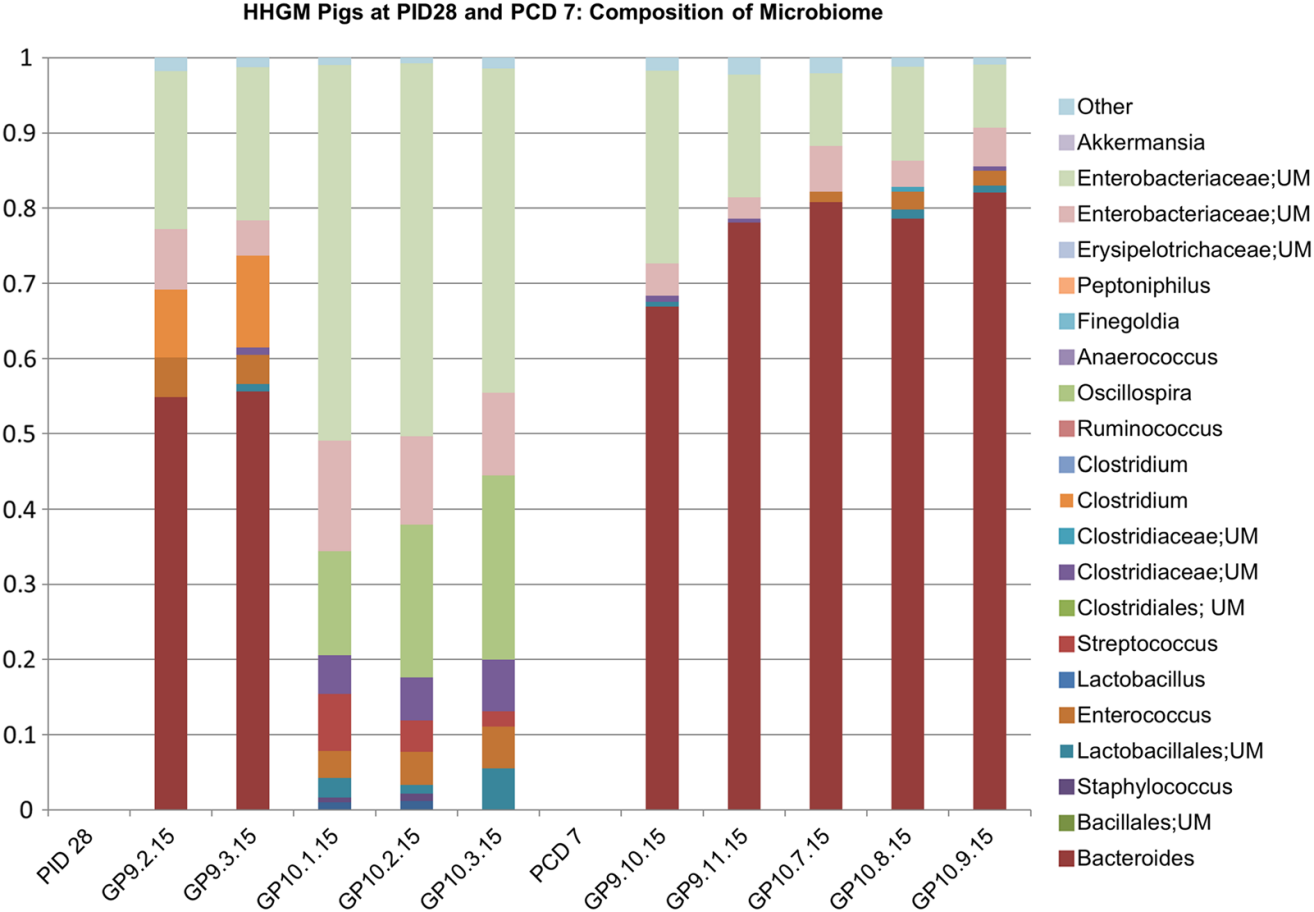
Relative abundance of specified bacterial taxa in the microbial community of large intestinal contents in HHGM pigs. Relative abundance of order, family, or genera of bacteria in the large intestinal contents at specified time points in HHGM pigs. *Bars* to the *right* of PID 28 are pigs euthanized on PID 28. *Bars* to the *right* of PCD 7 are pigs euthanized on PCD 7

**Fig. 7:**
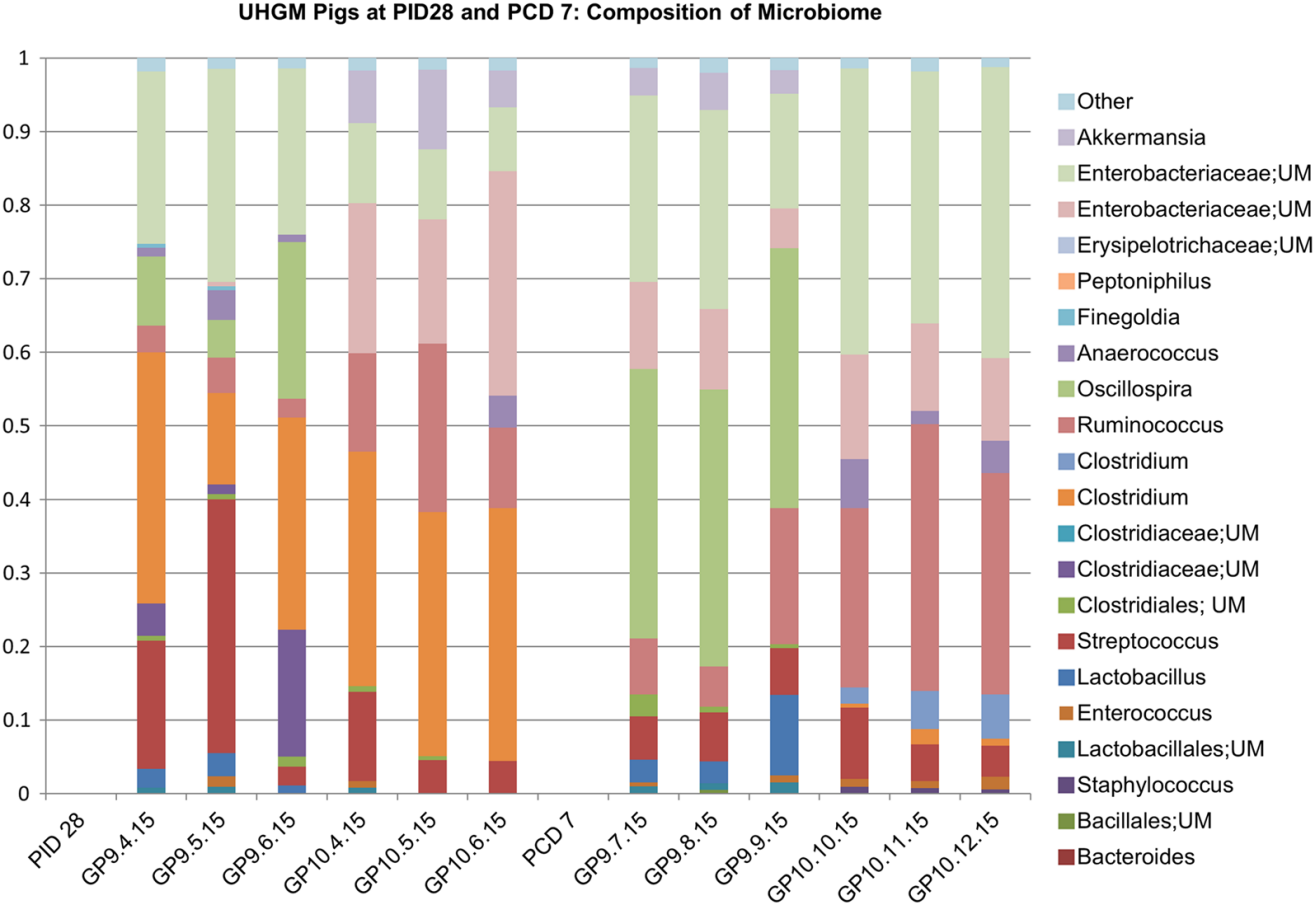
Relative abundance of specified bacterial taxa in the microbial community of large intestinal contents in UHGM pigs. Relative abundance of order, family, or genera of bacteria in the large intestinal contents at specified time points in UHGM pigs. *Bars* to the *right* of PID 28 are pigs euthanized on PID 28. *Bars* to the *right* of PCD 7 are pigs euthanized on PCD 7

Differences at the genera level were also evaluated between the two groups. On PID28, the mean relative abundance was significantly higher for the following OTUs in the HHGM group compared to the UHGM group; *Enterococcus*, *Collinsella*, *Stenotrophomonas*, *Pseudomonas*, and unclassified members of Lactobacillales and Enterococcaceae. The mean relative abundance of the following OTUs were significantly higher in UHGM pigs compared to HHGM pigs on PID28; *Clostridium, Streptococcus*, *Ruminococcus*, *Anaerococcus*, *Propionibacterium, Blautia*, and unclassified members of Clostridiales, Bacillales, and Lachnospiraceae (Figs. 6, 7).

At PCD7, mean relative abundance was significantly higher for *Bacteroides, Collinsella,* and unclassified members of Clostridiaceae and Erysipelothichaceae in HHGM pigs compared to that in UHGM pigs. UHGM pigs had a significantly higher mean relative abundance of *Ruminococcus*, *Streptococcus*, *Clostridium*, *Bifidobacterium*, *Staphylococcus*, *Turicibacter, Propriobacterium, Haemophilus*, *Moraxella*, *Blautia*, *Prevotella*, *Granulicatella,* and unclassified members of Enterobacteriaceae, Bacillales, Lachnospiraceae, and Clostridiales than the HHGM pigs (Figs. 6, 7).

Spearman’s correlation coefficients were determined for frequencies of rotavirus-specific IFN-γ producing T cells in ileum, spleen, and blood, and OTUs at the genus level on PID28 (PCD0) and PCD7 (Table 3). There were significant positive correlations between *Collinsella* and CD8+ T cells in blood and ileum, as well as CD4+ T cells in blood at PCD0. At PCD0, significant negative correlations existed between *Clostridium* and *Anaerococcus,* and ileal CD8+ and CD4+ T cells. At this time point, CD8+ T cells in blood were negatively correlated with *Propionibacterium*, *Blautia,* and an unclassified member of Bacillales, while CD4+ T cells in blood were negatively correlated with 2 unclassified members of Clostridiales.

**Table 3:** Spearman’s rank correlation coefficients between specified OTUs and rotavirus-specific IFN-γ+CD8+ or IFN-γ+CD4+ T cells among all Gn pigs

	OTU	Tissue	T cell type	ρ	*p* value	adj. *p* value
Positive correlations						
PCD0	*Collinsella*	Ileum	CD8+	0.91	<0.01	0.001
	*Collinsella*	Blood	CD8+	0.83	<0.01	0.016
	*Collinsella*	Blood	CD4+	0.89	<0.01	0.002
PCD7	Clostridiales (unclassified)	Ileum	CD8+	0.89	<0.01	0.002
	Mycoplasmataceae (unclassified)	Ileum	CD8+	0.80	0.01	0.021
Negative correlations						
PCD0	*Clostridium*	Ileum	CD8+	−0.90	<0.01	0.002
	*Anaerococcus*	Ileum	CD8+	−0.88	<0.01	0.002
	*Propionibacterium*	Blood	CD8+	−0.88	<0.01	0.002
	Bacillales (unclassified)	Blood	CD8+	−0.87	<0.01	0.003
	*Blautia*	Blood	CD8+	−0.81	<0.01	0.009
	*Clostridium*	Ileum	CD4+	−0.83	<0.01	0.013
	*Anaerococcus*	Ileum	CD4+	−0.86	<0.01	0.006
	Clostridiales (unclassified)	Blood	CD4+	−0.89	<0.01	<0.01
	Clostridiales (unclassified)	Blood	CD4+	−0.82	<0.01	0.014
PCD7	*Anaerococcus*	Ileum	CD8+	−0.84	<0.01	0.010
	*Anaerococcus*	Spleen	CD8+	−0.83	<0.01	0.016
	*Anaerococcus*	Spleen	CD4+	−0.87	<0.01	0.005
	*Staphylococcus*	Spleen	CD4+	−0.81	<0.01	0.020

At PCD7, there were significant positive correlations between unclassified members of Clostridiales and *Mycoplasmataceae* and ileal CD8+ T cells. Splenic CD8+ and CD4+ T cells and ileal CD8+ T cells were negatively correlated with *Anaerococcus*. A negative correlation also existed between splenic CD4+ T cells and Staphylococcus.

These data suggest the influence of specific bacterial OTUs on the vaccine-induced T cell responses, as well as the selective impact of IFN-γ producing T cell responses on the gut microbiome. Further investigations are needed to determine which OTUs are most important for influencing the immune response and the mechanism by which they do so.

### Enteropathy biomarkers, histopathology, pig weights

Concentrations of α-1-antitrypsin, myeloperoxidase and regenerating islet derived protein 1 beta (REG1B) in SIC and LIC were measured using porcine specific ELISAs. Alpha-1-antitrypsin is a serum protein that is not present in stool unless there increased gut permeability [9]. Myeloperoxidase is released from activated neutrophils and is an indicator of inflammation [9]. REG1B is a pro proliferative antiapoptotic protein secreted by damaged enterocyte and is involved in tissue repair, cell growth and regeneration [23, 24]. There were no significant differences between the two pig groups at PID28 or PCD7 (Additional file 1: Table S2). When compared to those of the two human infants (Table 4), the α-1-antitrypsin and myeloperoxidase concentrations were even lower than the HHGM, suggesting that no enteropathy developed in the pigs.

**Table 4:** Characterization of HGM samples used for oral inoculation of Gn pigs

	SV14 (healthy gut)	PM25 (unhealthy gut)
Enteropathy score	4	11
Myeloperoxidase	0.37 μg/ml	2.08 μg/ml
α-1-antitrypsin	22.9 μg/ml	141.4 μg/ml
Neopterin	74.7 nmol/l	412.4 nmol/l
Calprotectin	148.5 μg/g	220.6 μg/g
Pre-vaccination IgA titer	1:20	1:50
Post-vaccination IgA titer	1:3200	1:100
Fold increase in IgA titer	160	2
Phylogenetic diversity	7.8	5.7
Shannon Index	4	3.3
Observed species	107.4	67.3

Histopathologic evaluation of villus length, crypt depth, villus to crypt ratio, villus width, and mitotic index in duodenum, jejunum, and ileum did not reveal any significant differences between the two groups at the two time points (Additional file 1: Table S3; Additional file 2: Figure S1, Additional file 3: Figure S2, Additional file 4: Figure S3). Thus, the enteropathy status of the UHGM infant donor, as indicated by the higher ES of the UHGM samples, was not recapitulated in the UHGM-colonized Gn pigs.

Weights of pigs did not differ between the groups at any of the time points (Additional file 1: Table S4). This suggests the microbiota did not differentially affect nutrient assimilation or growth. The lack of significant differences between the groups in regards to enteropathy biomarkers, histopathology, or weight indicates enteropathy did not develop in this Gn model within the timeframe of this experiment.

## Discussion

In this study we demonstrated that Gn pigs colonized by UHGM can serve as a model system for enteric dysbiosis and impaired immunity. We used this neonatal pig model system to evaluate the effects of gut microbiota on immune responses to oral rotavirus vaccination. We also evaluated the impact of rotavirus challenge on the gut microbiota. In this model system, the only variable was the different HGM; thus any observed difference in the results could be attributed to differences in the gut microbiota. We demonstrated that pigs colonized with HHGM had a significantly stronger effector T cell immune response to vaccination compared to UHGM pigs. Additionally, we found significant correlations between some bacterial OTUs and frequencies of effector T cells, and we also observed differences in the gut microbiome composition and diversity both before and after VirHRV challenge.

When the two groups were compared, UHGM had weaker virus-specific T cell immune responses to AttHRV and trended lower rotavirus-specific antibody titers in intestinal contents. These data indicate that HHGM facilitated a stronger adaptive immune response to oral rotavirus vaccine than did UHGM. Evidently, UHGM pigs had higher viral shedding and more severe clinical signs compared to HHGM pigs after challenge with VirHRV.

The rotavirus challenge caused significant decreases in alpha diversity indices in the HHGM pigs, while no significant changes were detected in UHGM pigs. The decreased alpha diversity in HHGM pigs after VirHRV challenge was expected. A study evaluating microbiome and diarrhea found that diarrhea was associated with decreased phylogenetic diversity [25]. Although no samples were available for time points after PCD7, it is expected, given our data on enteropathy biomarkers, that diversity of HHGM pigs would return to normal levels. The lack of significant differences in alpha diversity parameters in UHGM pigs from PID28 to PCD7 may be due to an already abnormal microbiome and/or previous exposure to rotavirus based on pre-vaccination serum IgA titer in the infant donor.

The mean relative abundance of phyla between UHGM pig LIC samples and infant stool sample (PM25) was similar, which was as expected. However, HHGM pig samples differed from their infant donor (SV14). Community structure may have been different between the human and pigs because of influence of different environments, differences in diet, lack of natural microbial succession in the pigs, or differences in host genetics [26]. The phylum and genus level differences between HHGM and UHGM pigs at both time points warrant further study to assess the influences of these OTUs on the host immune response. In HHGM pigs, after VirHRV challenge, there was a decrease in the relative abundance of Firmicutes, similar to previous observations [21]. Although we did not sample pigs without AttHRV vaccination, human studies have shown that rotavirus vaccination does not have any major effects on the gut microbiota of children [27, 28]. Similar to a human study, HHGM pigs had an increased mean relative abundance of *Bacteroides* after rotavirus infection [29]. *Bacteroides* and *Lactobacillus* species have been shown to modify cell-surface glycans in human intestinal cultured cells, effectively blocking rotavirus infection [30]. This may partially explain why HHGM pigs had decreased viral shedding when compared to UHGM pigs. In agreement with a previous study, we also observed a decrease in levels of *Streptococcus* in HHGM pigs after the rotavirus challenge [21]. There is limited data on mechanisms by which microbiota directly influences enteric virus infectivity. Microbiota may modify the cell surface or bind to pathogens. *In vitro* experiments have demonstrated soluble factors from *Bacteroides thetaiotaomicron* and *Lactobacillus casei* can increase cell surface galactose and block rotavirus infection [30]. Poliovirus binds bacterial surface polysaccharides, which enhances virion stability and cell attachment, and may enhance transmission [31]. Gnotobiotic pigs colonized with *E*. *coli* Nissle 1917 (EcN) had lower viral shedding titers, which the authors speculated was because EcN bound to HRV particles [32].

There were significant positive and negative correlations between OTUs and IFN-γ producing T cell responses in the ileum, blood, and spleen. The biological relevance of these findings needs to be explored further. Previous studies have shown that the HGM promotes development of the neonatal immune system as evidenced by the significantly enhanced IFN-γ producing T cell response and decreased regulatory T cells in AttHRV-vaccinated pigs when comparing HGM colonized Gn pigs to non-HGM colonized Gn pigs [20]. Human studies have shown correlations between microbiome components and response to vaccination. In Bangladeshi infants, Actinobacteria, Coriobacteriaceae, and *Bifidobacterium* abundance were positively correlated with T cell responses to oral polio vaccine, while Pseuodmonadales, Clostridiales, and Enterobacteriales had a negative correlation [33]. Concurring with the human study, in this present study *Collinsella,* a member of the Coriobacteriaceae, was strongly and positively correlated with intestinal and circulating rotavirus specific IFN-γ producing CD8+ T cell responses, which are known to correlate with protection against rotavirus diarrhea [18].

We successfully generated a model of dysbiosis, but not enteropathy. There were no differences between the pig groups in regards to gut permeability, assessed by α−1-antitrypsin, gut inflammation, assessed by myeloperoxidase or epithelial regeneration assessed by REG1B. Histopathologic parameters did not differ between the groups. Our Gn pig model did not exhibit small intestinal villous blunting, crypt hyperplasia, or lymphocytic infiltration, which are the histologic lesions of EED. The pigs also did not have microscopic lesions of rotavirus infection, the lack of which may be partially due to timing. At PCD7, the small intestine has recovered from rotavirus infection and there are no histopathologic lesions in Gn pigs [15]. Although the nutritional status of the pigs was not assessed in this study, the lack of significant differences in body weights between the groups suggests that nutrient assimilation was not altered. We can speculate that other factors such as malnutrition or concurrent enteropathogen infections are needed to induce the EED phenotype. Additionally, 6 weeks may not have provided sufficient time to develop EED; however it is logistically difficult to keep Gn pigs for extended periods of time.

A recent paper describing a Gn mouse model of EED demonstrated that both malnutrition and gut microbiome were key to developing the model [34]. Our pigs received a nutritionally adequate diet. Thus, we suspect incorporation of malnutrition, and potentially enteropathogens into our dysbiosis model will be required to establish a Gn pig model of EED. A mouse study demonstrated that protein-energy malnutrition alone does not impair vaccine efficacy or increase the severity of infection [35]. Another recent paper showed that EED, systemic inflammation, and poor maternal health were associated with underperformance of oral rotavirus vaccine (Rotarix®) and oral poliovirus vaccine but not parenteral vaccines in Bangladeshi children [11]. Systemic inflammation and poor maternal health were also predictive of malnutrition [11]. In the future, we can use the Gn pig model to address effects of malnutrition and systemic inflammation on gut immunity.

In future studies, if we succeed in creating histologic changes and altered biomarker concentrations in our attempt to create an enteropathy pig model, additional assays can be utilized to further characterize the model. Tight junction and adherens junction proteins such as occludin, claudins, E-cadherin, and catenins can provide further insight into intestinal permeability. Systemic inflammation can be assessed with cytokines and acute phase proteins such as IL-1β, IL-4, IL-5, TNFα, C reactive protein (CRP), ferritin, and soluble CD14 (sCD14) [11]. Endotoxin core antibody IgG (Endocab) in serum can be used to evaluate bacterial translocation as well as systemic inflammation [9]. The lactulose to mannitol ratio assay is a standard test used in children to assess intestinal permeability and impaired tight junctions [9]. We did not utilize this test as the requirement for 2–5 h urine collection and the need to use a closed urinary collection system in the pigs is not technically feasible with gnotobiotic pig isolators or behavior of pigs [9].

There are limitations to this study. Due to the size and weight constraints of the pig isolators, long-term studies are not feasible. This limitation did not allow us to determine if structural or functional changes occur after a longer time period. Regarding the HGM, we used one HHGM and one UHGM sample, both from breastfed, vaginally delivered, Nicaraguan children living in homes with piped water and indoor toilets. As stated previously, numerous factors contribute to gut microbiome composition and environmental enteropathy. We do not know if the same results would be obtained with different HHGM and UHGM samples. Ideally, future studies will evaluate multiple HHGM and UHGM from children exposed to different variables such as diet, access to clean water, method of delivery and country of habitation with equal numbers of male and female samples. Despite the limitations, the Gn pig model of dysbiosis provides a valuable tool for future studies to investigate the immunomodulating mechanisms of the gut microbiota on the immune system and disease pathogenesis.

Further studies are needed to characterize the roles of specific bacterial species on enteric immunity and rotavirus infectivity. Additionally, by adding malnutrition and enteropathogens to this model, we may be able to recapitulate EED. A pig model of EED and rotavirus vaccination will be valuable since mouse models of subclinical enteric viral infections often do not predict vaccine efficacy against disease in humans. Rotavirus pathogenesis and immunity are similar in pigs and humans, but different in mice [36].

## Conclusions

We established a HGM transplanted Gn pig model of dysbiosis and evaluated the influence of the gut microbiota on vaccine immunogenicity and microbiota response to VirHRV challenge. We demonstrated that impaired enteric immunity in human infants can be recapitulated in the Gn pig model with UHGM transplantation. Our findings indicate that the gut microbiota has a major impact on vaccine immunogenicity. This animal model will be valuable in the evaluation of various strategies (i.e. probiotics, prebiotics, nutritional supplementation) to modulate the intestinal microbiome in order to enhance the immune responses to rotavirus vaccine as well as other vaccines, and enteropathogens such as norovirus, enterotoxigenic *E. coli* and *Shigella*. Results from this study provide a stepping stone for further research involving influence of specific bacterial species on enteric immunity and interaction with rotavirus. Additionally, by adding additional factors to this model such as malnutrition, it may be possible to emulate EED. Enteric dysbiosis, malnutrition, and EED interact in a complex and ill-defined way to negatively impact the health and immunity of young children in the developing world, leading to widespread morbidity and likely increased mortality.

## Methods

### Stool samples for HGM transplantation

**Fig. 8:**
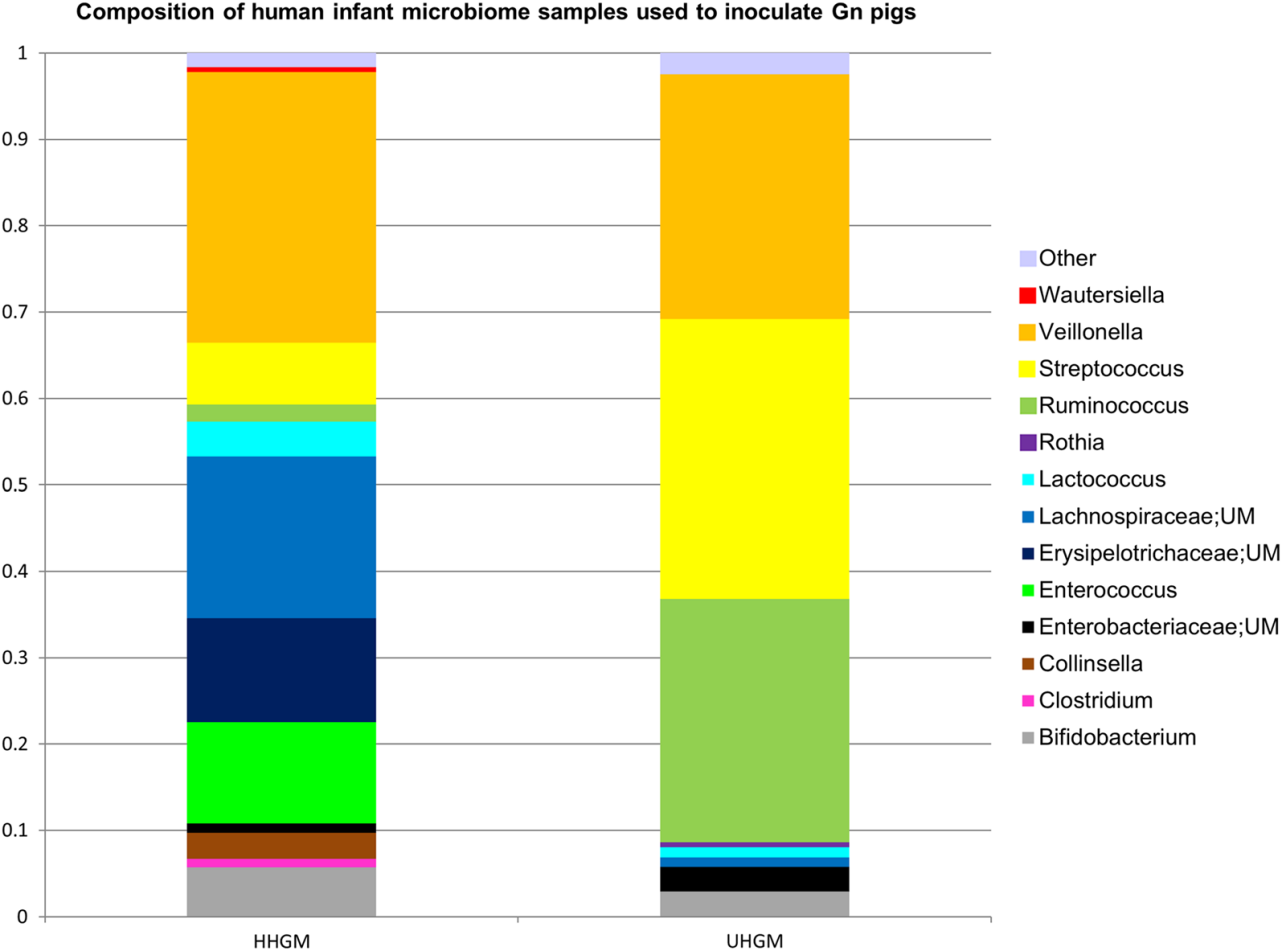
Composition of human infant microbiome samples used to inoculate Gn pigs. UM indicates unclassified member. Relative abundance is on the y-axis

Stool samples were obtained and analyzed from infants as described in a previous manuscript [37]. Briefly, infants from León, Nicaragua were recruited one day prior to their first pentavalent rotavirus (RV5) immunization, had blood drawn and a stool sample collected from a soiled diaper. A second blood sample was collected 1 month after the first dose of vaccine. Stool specimens were diluted 20-fold and homogenized in sterile pre-reduced anaerobic saline-0.1 M potassium phosphate buffer (pH 7.2) containing 15% glycerol (v/v). The samples were snap frozen in liquid nitrogen in the field, immediately transferred to −80 °C, shipped on dry ice to our lab, and stored at −80 °C until assayed. All stool samples underwent 16S rRNA amplicon sequencing for characterization of the gut microbiome, as previously described [25]. ELISAs for 4 biomarkers of enteropathy (α−1 antitrypsin, neopterin, myeloperoxidase and calprotectin) were run on the samples [38, 39]. Results from each marker were divided into quantiles with 1 assigned to first quantile, 2 for second quantile, 3 for third quantile and 4 for fourth quantile. The numbers were then added to create a combined enteropathy score (ES) for these 4 biomarkers ranging from 4 to 16. Rotavirus-specific serum IgA titers were measured on all blood samples. Results of the infant studies were detailed in a previous publication [37]. Compared to infants who seroconverted, infants who did not seroconvert had higher concentrations of both myeloperoxidase and calprotectin in stool samples, and higher mean ES. The UHGM sample (ID PM25) was collected from a 9-week old female child with poor seroconversion to the RV5 vaccine, high ES and low alpha diversity (AD). Conversely, the HHGM sample (ID SV14) was collected from an 8-week old male child with a strong IgA response to the RV5 vaccine, low ES, and high AD. The parameters for each variable in the two samples are listed in Table 4. The composition of the microbiome for each sample is shown in Fig. 8. Both children were delivered vaginally and breastfed. They came from different homes; both homes had municipal indoor piped water and indoor toilets.

Studies were approved by the Office of Human Research Ethics at the University of North Carolina (UNC) at Chapel Hill (#14-1136) and the Universidad Nacional Autónoma de Nicaragua, León (#110). Parents or legal guardians of the infants provided written consent.

### Selection and preparation of infant samples for HGM Gn pig transplantation

The UHGM stool sample was selected from an infant who did not seroconvert following receipt of the first dose of RV5 (seroconversion defined as >4-fold increase in rotavirus-specific serum IgA titers 1 month after RV5 immunization), low phylogenetic diversity of the gut microbiome, and high ES. The HHGM stool sample was selected from a child with high RV5 immunogenicity (experienced a >4-fold increase in rotavirus-specific serum IgA titers after RV5 immunization), high phylogenetic diversity of the gut microbiome, and low ES.

The selected HGM were screened and confirmed negative for rotavirus, astrovirus, norovirus, sapovirus, adenovirus, and *Klebsiella* spp. via PCR prior to oral transplantation into the Gn pigs. *Klebsiella* spp. were specifically targeted because previous studies have lost HGM transplanted pigs to *Klebsiella* infection from the inocula [20, 40]. Primers for the *Klebsiella* spp. PCR targeted the *gyr*A gene and were based on previous publications [41, 42]. The universal 16S rRNA primers, 27F and 1492R were included in the PCR protocol to confirm the presence of amplifiable DNA. DNA was extracted with ZR Fecal DNA MiniPrep (Zymo, Irvine, CA) from 150 µl of sample per manufacturer’s instructions. *Klebsiella pneumoniae*, obtained from the Virginia Tech Animal Laboratory Services bacteriology laboratory was used as a positive control sample. The master mix contained 5 µl PFX 50 PCR mix, 1 µl PFX 50 DNA polymerase (ThermoFisher Scientific, Waltham, MA), 1.5 µl 10 mM dNTPs, 1.5 µl of each primer (10 μM), 4–30 µl of DNA and water added to a final volume of 50 µl. After a 2-min incubation at 94 °C, 35 cycles of 94 °C 15 s, 60 °C 20 s, and 68 °C 1 min were performed, followed by a final extension at 68 °C for 5 min.

PCR for norovirus genogroups I and II, adenovirus, sapovirus, and astrovirus were carried out at St. Jude Children’s Research Hospital. DNA and RNA were extracted from 175 µl of sample using the MagMax Total Nucleic Acid isolation kit (ThermoFisher Scientific). PCR buffer for the RNA samples was Taqman fast virus 1-step (ThermoFisher Scientific). Taq core kit (Qiagen, Valencia, CA.) was used for DNA samples. Norovirus I and II, sapovirus, adenovirus, and astrovirus real-time PCR primers, as well as, probes and conditions, were based on previous publications [43–46].

RT-PCR for rotavirus gene segment 6 was used to screen samples for rotavirus. RNA was extracted from samples using TRIZOL LS (ThermoFisher Scientific) per manufacturer’s instructions. The mixture for cDNA synthesis included 2 µmol of primer 1581, and 11 µl of RNA. RNA was denatured at 95 °C for 5 min then cooled to 4 °C. The mixture for reverse transcription contained 4 µl of 5× buffer, 1 µl 0.1 M DTT, 1 µl Superscript III reverse transcriptase (ThermoFisher Scientific) and 1 µl of RNase free water. The cDNA sample was combined with the mixture for reverse transcription and incubated at 50 °C for 60 min then 70 °C for 15 min and held at 4 °C. One microlitre of RNaseH (New England Biolabs, Ipswich, MA) was added and the sample was incubated at 37 °C for 20 min. The PCR mix contained 10 µl of buffer, 1 µl of MyTaq DNA polymerase (Bioline, Taunton, MA), 27 µl of ddH2O, 1 µl of each 20 μM primer (158 (forward) and 15 (reverse), and 10 µl of DNA. The sequence for primer 158 was GGC TTT AAA ACG AAG TCT TCG AC and that for primer 15 was GGT CAC ATC CTC TCA CTA. The sample was incubated at 95 °C for 3 min followed by 35 cycles of 95 °C 20 s, 47.5 °C 30 s, and 72 °C 90 s. Final extension was at 72 °C for 7 min. AttHRV was used as a positive control.

HGM was prepared for oral inoculation into the Gn pigs as described previously [20]. The sample was washed with tenfold volume of sterile PBS to remove glycerol, and then centrifuged at 2000 rpm for 10 min at 4 °C. The pellet was resuspended to the original volume with sterile PBS. Pigs received 400–700 µl of HGM sample at 5–7 days of age.

### Pig studies

Near-term pigs (Yorkshire crossbred) were derived by hysterectomy and maintained in sterile isolator units as described previously [47]. The estimated sample size to obtain greater than 0.9 power is n ≥ 6 based on a power analysis using an ANOVA or ANCOVA model by the Virginia Tech Department of Statistics. Sterility was confirmed by culturing isolator swabs and porcine rectal swabs on blood agar plates and in thioglycollate media 3 days after derivation. Commercial ultra-high temperature-treated sterile cow milk was used throughout the study. The pigs were housed with a 12-h light–dark cycle at 80–93 °F (based on age) and provided with a toy for environmental enrichment. The animal experimental protocol is outlined in Additional file 1: Table S1. These pigs and data generated from them have not been used in previous publications. In total, 24 pigs were randomly assigned to the 4 groups (HHGM-PID28/PCD0 *n* = 5, HHGM PCD7 *n* = 7, UHGM PID28/PCD0 *n* = 6 and UHGM PCD7 *n* = 6). Intraperitoneal (IP) injections of gamma-irradiated, commercially available porcine serum (Rocky Mountain Biologicals Inc., Missoula, MT) were given to the pigs in an attempt to provide immunoprotection against potentially pathogenic bacteria in the HGM transplants. A total volume of 60 ml was given at 3 time points, 6 h apart, starting on the day of or day after derivation. In the commercial serum, rotavirus-specific virus neutralization titer was measured at 64, rotavirus-specific IgG titer at 65,536, and rotavirus-specific IgA titer at 64. At PCD 0, the geometric mean titers for rotavirus-specific antibodies among all pigs were 16.7 for virus neutralizing antibodies, 10,935 for IgG, and 75.9 for IgA. These titers mimic normal maternally acquired rotavirus antibody titers in conventionally derived suckling pigs or human infants [48]. The cell culture-adapted HRV Wa strain (G1P1A [8]), derived from the 35th passage in African green monkey kidney cells (MA104), was used as the AttHRV vaccine for oral inoculation at a dose of 5 × 10^7^ focus-forming units (FFU) on PID 0 (7 days of age), PID10 and PID20 [17]. A subset of pigs (*n* = 5–6) from each group was euthanized before challenge PID28/PCD0 for evaluation of immune responses induced by AttHRV vaccination. The remaining pigs (*n* = 6–7) were orally challenged with 10^5^ FFU of VirHRV Wa strain (G1P1A [8]) and monitored from PCD0-PCD7 to evaluate virus shedding and diarrhea before euthanasia on PCD7. The 50% infectious dose (ID50) of VirHRV in neonatal Gn pigs is approximately 1 FFU [15]. Rectal swabs were collected daily to monitor virus shedding by ELISA and CCIF from PCD0-PCD7. Daily diarrhea scoring was defined as (0) solid, (1) pasty, (2) semi-liquid, or (3) liquid. Scores of 2 or higher represented diarrhea.

All animal protocols were approved by the Institutional Animal Care and Use Committee at Virginia Tech (protocol #13-187-CVM) and performed in accordance with federal and university guidelines, as well as with recommendations in the American Veterinary Medical Association Guidelines for the Euthanasia of Animals. All sow surgeries were performed under ketamine and tiletamine-zolazepam anesthesia, and immediately followed by euthanasia. A lidocaine epidural was administered preoperatively.

### Rotavirus-specific serum and intestinal antibody titers

Pigs were bled and weighed weekly starting at 7 days of age (PID 0). The LIC and SIC were collected at necropsy. Determination of rotavirus-specific IgA and IgG antibody titers in serum and SIC, and IgA in LIC, were based on previously described methods [16, 49]. Briefly, 96-well microtiter plates were coated with semi-purified AttHRV antigen at 1:60 or mock-infected MA104 cell culture control lysate at 1:60 in 0.05 M carbonate buffer pH 9.6 overnight at room temperature. The plate was washed with TBS pH 8.0 with 0.05% Tween (TBST) five times. Plates were blocked with TBS containing 1% BSA for 1 h at room temperature. Four-fold serial dilutions of each sample in TBST containing 1% BSA were performed on a separate plate. The samples were then transferred to the antigen-coated plate and incubated for 2 h at room temperature followed by five washes with TBST. Plates were then incubated for 1 h at room temperature with the detection antibody. The IgA assay used goat anti-porcine IgA polyclonal HRP-conjugated antibody (A100-102P, Montgomery, TX; AB_67219) and the IgG assay used goat anti-porcine IgG-Fc fragment polyclonal HRP-conjugated antibody (A100-104P, Bethyl Laboratories, Inc.; AB_67225). Both antibodies were diluted 1:35000 in TBST containing 1% BSA. Plates were washed five times with TBST and developed with ABTS (2,2′-azino-di(3-ethylbenzothiazoline-6-sulfonate) HRP substrate (KPL, Inc., Gaithersburg, MD) for 15–30 min at room temperature. The reaction was stopped with ABST peroxidase stop solution (KPL, Inc.) diluted 1:5 in water. Optical density was measured at 405 nm.

### Rotavirus-specific serum virus neutralizing antibody titer

African green monkey kidney cells, MA104 (ATCC, Manassas, VA, # CRL-2378.1, purchased from ATCC in January 2008) were grown in 96-well plates for 3–4 days. Monolayers were washed once with Eagle’s minimum essential medium (EMEM) (ATCC), and 100 µl of EMEM was added to each well. The plate was incubated at 37 °C for 2 h. In a separate 96-well plate, serum samples were diluted 4-fold in duplicate. One set of each sample was inoculated with AttHRV (4 × 10^3^ FFU) and the other set with EMEM. This plate was incubated at 37 °C for 1 h. After the 2 h incubation, EMEM in the MA104 plates was discarded and the cells were incubated with 50 µl/well of the serum with AttHRV or EMEM in duplicate at 37 °C for 1 h. Fifty microlitre of EMEM with trypsin (0.5 μg/ml) was added to each well and the plate was incubated at 37 °C with 5% CO_2_ for 18–24 h. Liquid in the wells was discarded and the plates were fixed with 80% acetone at room temperature for 10 min. Acetone was discarded and plates were air-dried. Fixed plates were stored at −20 °C or stained immediately. For immunofluorescence staining, plates were initially rinsed with PBS containing 0.05% Tween 20 (PBST) pH7.4 once for 2 min. Each well received 50 µl of goat anti-bovine rotavirus polyclonal unconjugated antibody (PA1-7241, ThermoFisher Scientific; AB_561090) diluted 1:250 in PBST containing 2% nonfat dry milk then incubated at 37 °C for 1 h. Plates were rinsed 3 times with PBST. Fifty microlitre of rabbit anti-goat IgG whole molecule polyclonal FITC-conjugated antibody (F7367, Sigma-Aldrich, St. Louis, MO; AB_259726) diluted 1:400 in PBST containing 2% nonfat dry milk was added to each well and incubated at 37 °C for 1 h. Plates were rinsed 3 times with PBS pH 7.4 and once with PBS pH8.0. Mounting media (60% glycerol, 40% PBS pH 8.0) was added to each well. Fluorescing cells were enumerated by fluorescent microscopy. The virus neutralizing antibody titer was defined as the reciprocal of the serum dilution which reduced the number of fluorescent cell forming units by >80% [50].

### CCIF and rotavirus antigen ELISA

Rectal swabs were processed as reported in a previous study [51]. Briefly, two rectal swabs per pig were rinsed in 8 ml diluent #5 (minimal essential media (MEM, ThermoFisher Scientific) 1% penicillin and streptomycin, 1% HEPES) and then centrifuged at 2100 rpm for 15 min at 4  °C. Viral antigen and infectious virus in the rectal swabs were measured using ELISA or cell culture immunofluorescence (CCIF), respectively, as previously described [52].

The CCIF was performed in MA104 cells grown in 96-well plates for 3–4 days. Plates were washed with PBS. One hundred microlitre of EMEM was added to each well and the plate was incubated for 2 h at 37  °C with 5% CO_2_. In separate 96-well plates, tenfold serial dilutions of the samples were made in EMEM. After the plate with cells was removed from the incubator, media was discarded. Fifty microlitre from each well on the dilution plate was added to the cells in duplicate. The plate was centrifuged at 2000 rpm for 1 h at 21 °C with slow deceleration. EMEM containing trypsin was then added to each well for a final trypsin concentration of 0.5 µg/ml. The cells were incubated at 37 °C for 18–24 h in a CO_2_ incubator. The protocol for acetone fixation and immunofluorescence staining of the plates was the same as described for the neutralizing antibody titer assay.

Using ELISA to detect rotavirus antigen, a 96-well microtiter plate was coated with 100 µl/well goat anti-bovine rotavirus polyclonal unconjugated antibody (PA1-7241, ThermoFisher Scientific; AB_561090) in 0.05 M carbonate buffer pH 9.6 at a dilution of 1:250. The plates were incubated overnight at 4  °C. Plates were then washed twice with PBST, blocked with 300 µl/well of PBS pH 7.4 containing 5% nonfat dry milk then incubated for 1 h at 37  °C. Plates were washed 3 times with PBST. One hundred microlitre/well of diluted sample (in PBS with 0.1% BSA) was added in duplicate. Semi-purified AttHRV antigen or supernatant of mock-infected MA104 cells were used as a positive and negative control respectively. Plates were incubated for 1 h at 37  °C then washed 3 times with PBST. One hundred microlitre/well of goat anti-bovine rotavirus polyclonal HRP-conjugated antibody (PA1-73015, ThermoFisher Scientific; AB_1018382) diluted 1:200 in PBS containing 1% BSA was then added to each well and incubated for 1 h at 37  °C, followed by 3 washes with PBST. One hundred microlitre/well of ABST peroxidase substrate solution was added and the plate was incubated for 15–30 min at room temperature. The reaction was stopped with 100 µl/well of ABST stop solution diluted 1:5. The optical density values were measured at 405 nm.

### Mononuclear cell isolation

Mononuclear cells (MNC) were isolated from the ileum, spleen, and blood as described in previous publications [17, 18, 53]. Briefly, after rinsing the ileum samples with HBSS without Ca^2+^ and Mg^2+^ (GE Healthcare, Logan, UT), they were manually minced with scissors, resuspended in 40 ml solution A (HBSS without Ca^2+^, Mg^2+^ with 200 μg/ml gentamicin, 20 μg/ml ampicillin, 40 mM HEPES, 5 mM EDTA, 0.29 mg/ml DTT, and 7% NaHCO3) then placed on a shaker at 37 °C for 30 min. Tissue fragments were further minced and solution C (RPMI-1640 (ThermoFisher Scientific) with 8% FBS, 200 μg/ml gentamicin, 20 μg/ml ampicillin, 20 mM HEPES, 5 mM EDTA, 0.29 mg/ml DTT, and 400 U/ml collagenase type II (Worthington Biochemical Co, Lakewood, NJ) was added to 40 ml and digested at 37  °C for 30 min with shaking. Supernatants were collected and remaining tissue was ground on an 80–mesh screen. The cell suspension was collected and the remaining tissue underwent another digestion step with repeated grinding on the screen. Cell suspensions were divided equally between three 50 ml conical vials and wash media (RPMI-1640 with 200 μg/ml gentamicin, 20 ug/ml ampicillin, and 10 mM HEPES) was added to a volume of 35 ml. Percoll (GE Healthcare) was added to each tube to bring the final Percoll concentration to 30% and then centrifuged at 1800*g* for 20 min at 4  °C without the brake. Cell pellets were resuspended in 15 ml conical vials with 10 ml of 43% Percoll, underlaid with 5 ml of 70% Percoll, and centrifuged at 1800*g* for 30 min at 4  °C without brake. MNC were collected from the 43 to 70% Percoll interface into 50 ml conical vials with 50 ml of wash buffer and centrifuged at 800*g* for 15 min at 4 °C.

Blood was mixed with 30% (vol/vol) of acid citrate dextrose at the time of necropsy, divided equally between three 50 ml conical vials with HBSS added to bring the volume to 50 ml, and then centrifuged for 30 min at 1200*g* with no brake at room temperature. The buffy coat was transferred to a 50 ml conical vial with HBSS added to a volume of 30 ml. The buffy coat mixture is then divided equally among three 15 ml centrifuge tubes, underlaid with 5 ml of Ficoll-Paque PREMIUM® (GE Healthcare Bio-Sciences, Pittsburgh, PA) and centrifuged at 1200*g* for 30 min at room temperature with no break. MNC were collected from the interface, placed in a 50 ml vial, HBSS added to bring the volume to 30 ml, and then centrifuged for 15 min at 800*g* 4 °C. Remaining erythrocytes were lysed by mixing 5 ml of sterile distilled water with the cell pellet, after which HBSS was added to a final volume of 50 ml. The samples were centrifuged for 15 min at 400*g* 4 °C, after which the HBSS wash and centrifugation step was repeated.

The spleens were minced and ground as described for the ileum without the solution C step. Cells were suspended in 50 ml of wash media then centrifuged for 30 min 1000*g* 4 °C. The cell pellet was resuspended in 20 ml of wash buffer and then Percoll was added to bring the final Percoll concentration to 30%. Samples were centrifuged for 1200*g* 4 °C without brake. Cells were resuspended with 30 ml of 43% Percoll, divided into three 15 ml tubes, underlaid with 5 ml of 90% Percoll and then centrifuged for 30 min at 1900*g* for 30 min 4 °C without the brake. MNC were collected at the 43–70% Percoll interface into a 50 ml conical vile with wash buffer, centrifuged for 15 min at 504*g* for 15 min 4 °C twice, changing the wash buffer between steps. MNC from all tissues were resuspended with 2–10 ml of enriched-RPMI (RPMI-1640 with 20 mM HEPES, 100 μg/ml gentamicin, 10 μg/ml ampicillin, 8% FBS, 2 mM l-glutamine, 0.1 mM nonessential amino acids, 1 mM sodium pyruvate, and 50 mM 2-mercaptoethanol.

### Flow cytometry for rotavirus-specific IFN-γ producing T cells

Frequencies of HRV-specific IFN-γ producing CD4+ and CD8+ T cells among the lymphocytes of the ileum, spleen, and blood were determined with flow cytometry. MNC were stimulated in 12-well culture plates with 12 μg/ml of semi-purified AttHRV antigen for 17 h at 37 °C in a CO_2_ incubator. Brefeldin A (Sigma-Aldrich) at 5 mg/ml, and mouse anti-human CD49d monoclonal APC-conjugated antibody (561892, BD Biosciences, San Jose, CA; AB_10896134) at 1 µg/ml were added at hour 12 of the incubation. MNC were washed with staining buffer (3% FBS and 0.09% sodium azide in DPBS (0.2 mg/ml KCl, 0.2 mg/ml KH_2_PO_4_, 8 mg/ml NaCl and 2.16 mg/ml Na_2_HPO_4_·7H2O, pH 7.2–7.4) and centrifuged at 500*g* for 5 min 4 °C. The MNC (2 × 10^6^ cells/tube in 100 µl staining buffer) were stained at 4 °C for 15 min using the following monoclonal antibodies: (1) One microlitre mouse anti-porcine CD4a monoclonal FITC-conjugated antibody, clone 74-12-4 (559585, BD Biosciences; AB_397279), (2) 0.5 µl mouse anti-pig CD8α monoclonal SPRD-conjugated antibody, clone 76-2-11 (4520-13, SouthernBiotech, Birmingham, AL) and (3) 2 µl of mouse anti-pig CD3ε monoclonal UNLB-conjugated antibody, clone PPT3 (4510-01, Southern Biotech) and then washed with staining buffer. Next the MNC were centrifuged at 500*g* for 5 min, followed by the addition of 1 µl of rat anti-mouse IgG_1_ monoclonal APC-conjugated antibody, clone ×56 (550874, BD Biosciences; AB_398470) to the samples and stained for 15 min at 4 °C, and then washed with staining buffer. Samples were centrifuged at 500*g* for 5 min. One hundred microlitre of Cytofix/Cytoperm (BD Biosciences) was added to the samples, which were then incubated for 30 min at 4 °C and washed once with Perm/Wash buffer (BD Biosciences). Next, 1 µl of mouse anti-pig IFN-γ monoclonal PE-conjugated antibody, clone P2G10 (559812, BD Biosciences; AB_397341) was added and the samples were incubated at 4 °C for 30 min. The samples were washed with Perm/Wash buffer solution, centrifuged at 500*g* for 5 min and then had 0.25 ml of staining buffer added. A minimum of 100,000 events were acquired using a FACSAria sorter. Flow cytometry data were analyzed using FlowJo 7.2.2 software (Tree Star, Ashland, OR).

### DNA isolation and 16S rRNA amplicon sequencing

LIC were collected at necropsy, snap frozen in liquid nitrogen and shipped on dry ice to the UNC Microbiome Core Facility where 16S rRNA amplicon sequencing DNA isolation was carried out as described [25]. Briefly, samples (200 mg) were transferred to sterile 2 ml tubes containing 200 mg of 212–300 μm glass beads (Sigma-Aldrich) and 1.4 ml of Qiagen ASL buffer for bead beating and subsequently incubated at 95 °C for 5 min. Supernatants were treated with InhibiEx inhibitor adsorption tablets (Qiagen) to remove PCR inhibitors. After a brief centrifugation, supernatants were transferred to a new tube with Qiagen AL buffer containing Proteinase K (600 IU/μl) and incubated at 70 °C for 10 min. DNA was purified using Zymo-spin columns (Genesee Scientific, Morrisville, NC) and Qiagen buffers AW1 and AW2 as washing agents, and eluted in 10 mM Tris (pH 8.0).

Sequencing libraries were prepared from total DNA (12.5 ng) amplified using a combination (4:1) of Universal and *Bifidobacterium*-specific primers targeting the V1–V2 region of the bacterial 16S rRNA gene [54–56]. Primers contained overhang adapters for compatibility with the Illumina sequencing platform and sequencing adapters. Master mixes contained 12.5 ng of total DNA, 0.2 µM of each primer and 2× KAPA HiFi HotStart ReadyMix (KAPA Biosystems, Wilmington, MA). Amplicons were purified using the AMPure XP reagent (Beckman Coulter, Indianapolis, IN) and then amplified using a limited cycle PCR program, adding Illumina sequencing adapters and dual‐index barcodes [index 1(i7) and index 2(i5)] (Illumina, San Diego, CA) to the amplicon target. Final libraries were purified, quantified and normalized prior to pooling. The DNA library pool was then denatured with NaOH, diluted with hybridization buffer, and heat denatured before loading on the MiSeq reagent cartridge (Illumina) and on the MiSeq instrument (Illumina). Automated cluster generation and paired–end sequencing with dual reads were performed according to the manufacturer’s instructions.

### Sequencing data analysis

Multiplexed paired-end fastq files were generated from sequencing data using the Illumina software configureBclToFastq. The paired-end fastqs were then joined into a single multiplexed, single-end fastq using the software tool fastq-join. Demultiplexing and quality filtering was performed on the joined results and quality analysis reports were produced using the FastQC software. Subsequent analyses were performed using the Quantitative Insights Into Microbial Ecology (QIIME v.1.8.0) [57] as described [25, 58]. Sequences were clustered into operational taxonomic units (OTU) at 97% similarity threshold using UCLUST [59], and they were aligned in order to build a phylogenetic tree [60]. A random selection of 1118 sequences from each sample was used for rarefaction analysis to determine diversity using observed species (S) and phylogenetic diversity (PD) metrics on rarefied OTU tables. Beta diversity and principal coordinates analysis (PCoA) were calculated using unweighted UniFac distances between samples at a depth of 1118 sequences per sample. Taxa of the L6 OTU (genus level) table with a relative abundance of 0.5% or greater and all taxa of the L2 OTU (phylum level) table were compared between groups at PCD0 and PCD7. Alpha diversity was compared in a similar fashion. Spearman’s rank correlation coefficients were determined between the frequencies of T cells in the ileum, blood and spleen of all pigs and taxa of the L6 and L2 OTU tables at PCD0 and PCD7.

### Histopathology

Sections of duodenum, jejunum, and ileum were collected, routinely processed, and analyzed as previously described [51]. The pathologist, blinded to the animal identification, evaluated villus length, crypt depth, villus-to-crypt ratio, crypt mitotic index, and lamina propria width.

### Enteropathy biomarkers

Large intestinal contents collected at necropsy were used for biomarker evaluation using commercial porcine ELISA kits (α-1-antitrypsin, TSZ ELISA, BIOTANG Inc. Lexington, MA; Reg1B, Elabsceince, Wuhan, Hubei, China; myeloperoxidase, MyBioSource, San Diego, CA). Assays were run per manufacturer’s instructions.

### Statistical analysis

Kruskal–Wallis rank sum test was used for comparisons of rotavirus-specific antibody titers in serum, LIC and SIC, frequencies of IFN-γ producing T cells, enteropathy biomarkers measured in pigs, histopathology, diarrhea duration and score, virus shedding duration and titer, and comparisons of microbiome composition between HGM groups. Fisher’s exact test was used for comparisons of percentages of virus shedding and diarrhea. The correlations between T cell frequencies in the ileum, blood and spleen of all pigs, and taxa of the L6 OTU table at PCD0 and PCD7 were determined by Spearman’s rank correlation analysis with the Benjamini-Hochberg method for false detection rate. Correlations were considered strongly negative or positive if ρ < −0.8 or ρ > 0.8, respectively. Statistical significance was assessed at *p* < 0.05.

## Additional files

**Additional file 1: Table S1.** Experimental design for Gn pig study. **Table S2.** Mean concentration of specified enteropathy biomarkers in large intestinal contents on PID28 and PCD7. **Table S3**. Mean measurements per group for specified parameter and tissue. V:C is the villous to crypt ratio. Mitotic index was determined by counting mitotic figures in 50 crypts. Measurements are given in mm. **Table S4.** Weights (in kg) of Gn pigs during the study.

**Additional file 2: Figure S1.** Duodenum Histopathology. Representative photomicrographs of duodenum sections from a pig in each group at each time point. A. HHGM PID 28, B. HHGM PCD 7, C. UHGM PID28, D. UHGM PCD7. There are no significant histologic differences between the groups. Scale bar is 200 μm.

**Additional file 3: Figure S2.** Jejunum Histopathology. Representative photomicrographs of jejunum sections from a pig in each group at each time point. A. HHGM PID 28, B. HHGM PCD 7, C. UHGM PID28, D. UHGM PCD7. There are no significant histologic differences between the groups. Scale bar is 200 μm.

**Additional file 4: Figure S3.** Ileum Histopathology. Representative photomicrographs of ileum sections from a pig in each group at each time point. There are no significant histologic differences between the groups. A. HHGM PID 28, B. HHGM PCD 7, C. UHGM PID28, D. UHGM PCD7. Scale bar is 200 μm.
