# Supplementary figures and images for: Modeling human enteric dysbiosis and rotavirus immunity in gnotobiotic pigs
Source: Gut Pathog. 2016 Nov 8;8:51. doi: 10.1186/s13099-016-0136-y (PMC5100090; doi:10.1186/s13099-016-0136-y)

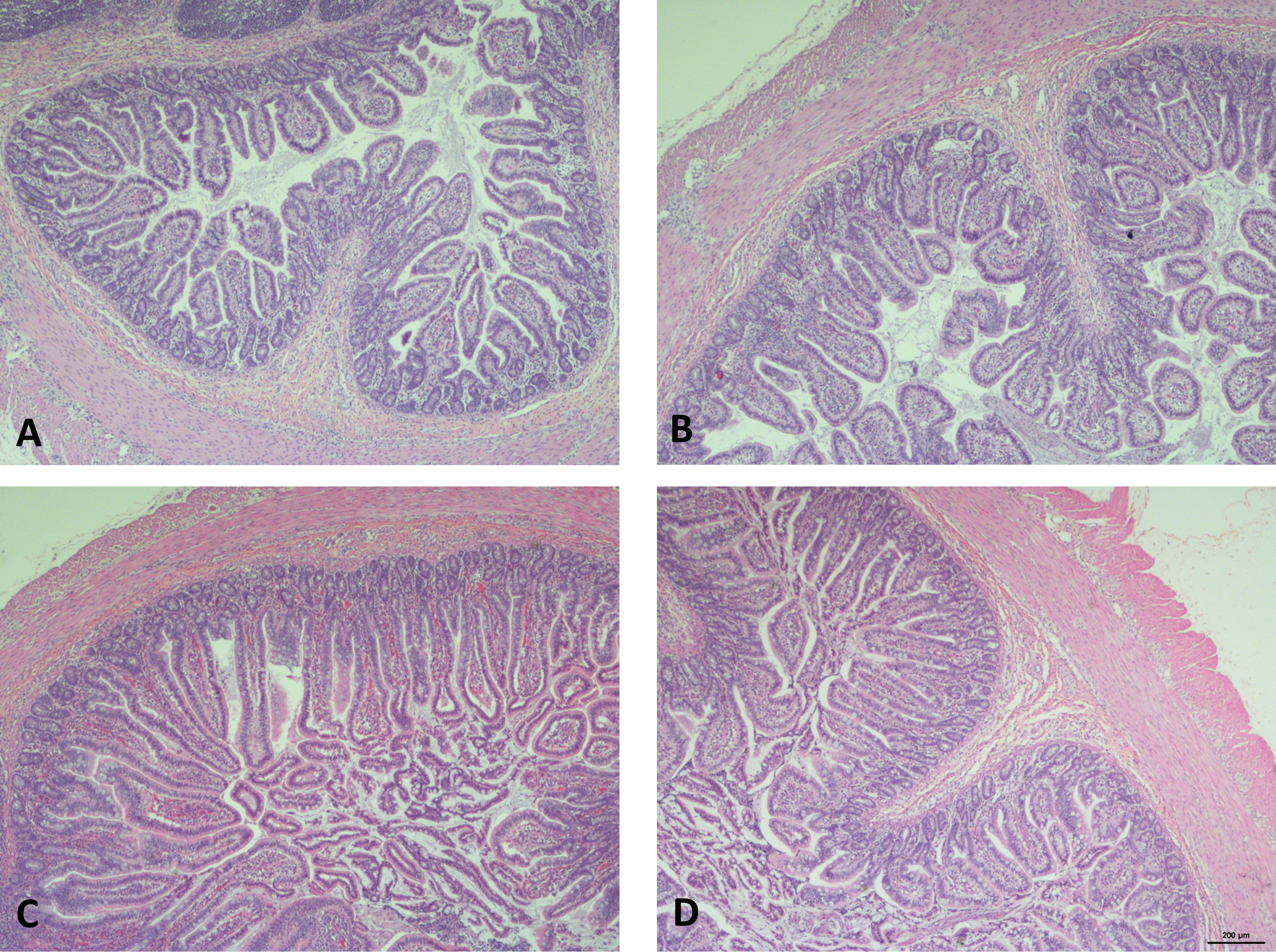

Supplement: Supplementary file 2 — Additional file 2: Figure S1. Duodenum Histopathology. Representative photomicrographs of duodenum sections from a pig in each group at each time point. A. HHGM PID 28, B. HHGM PCD 7, C. UHGM PID28, D. UHGM PCD7. There are no significant histologic differences between the groups. Scale bar is 200 μm. [file 13099_2016_136_MOESM2_ESM.tiff]

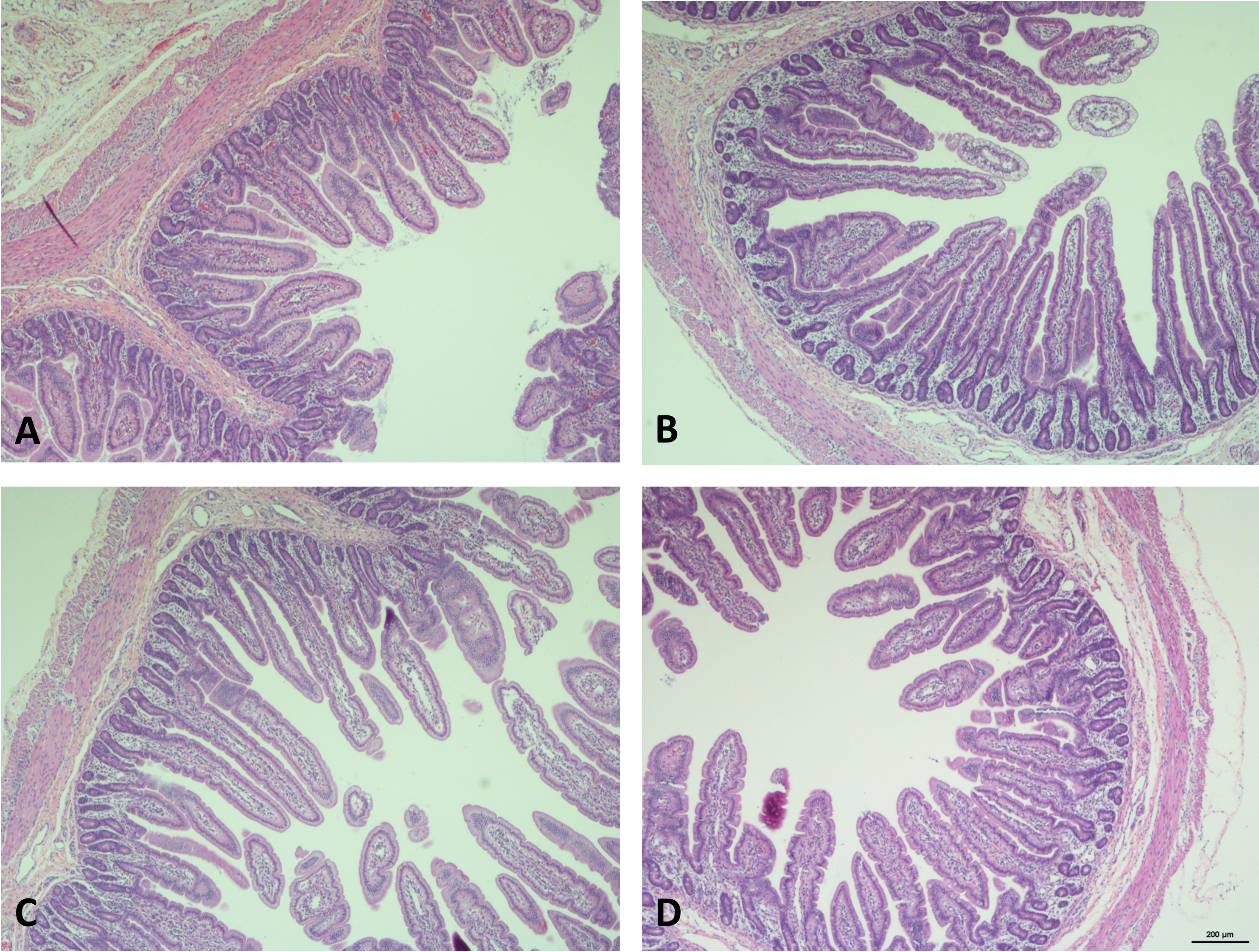

Supplement: Supplementary file 3 — Additional file 3: Figure S2. Jejunum Histopathology. Representative photomicrographs of jejunum sections from a pig in each group at each time point. A. HHGM PID 28, B. HHGM PCD 7, C. UHGM PID28, D. UHGM PCD7. There are no significant histologic differences between the groups. Scale bar is 200 μm. [file 13099_2016_136_MOESM3_ESM.tiff]

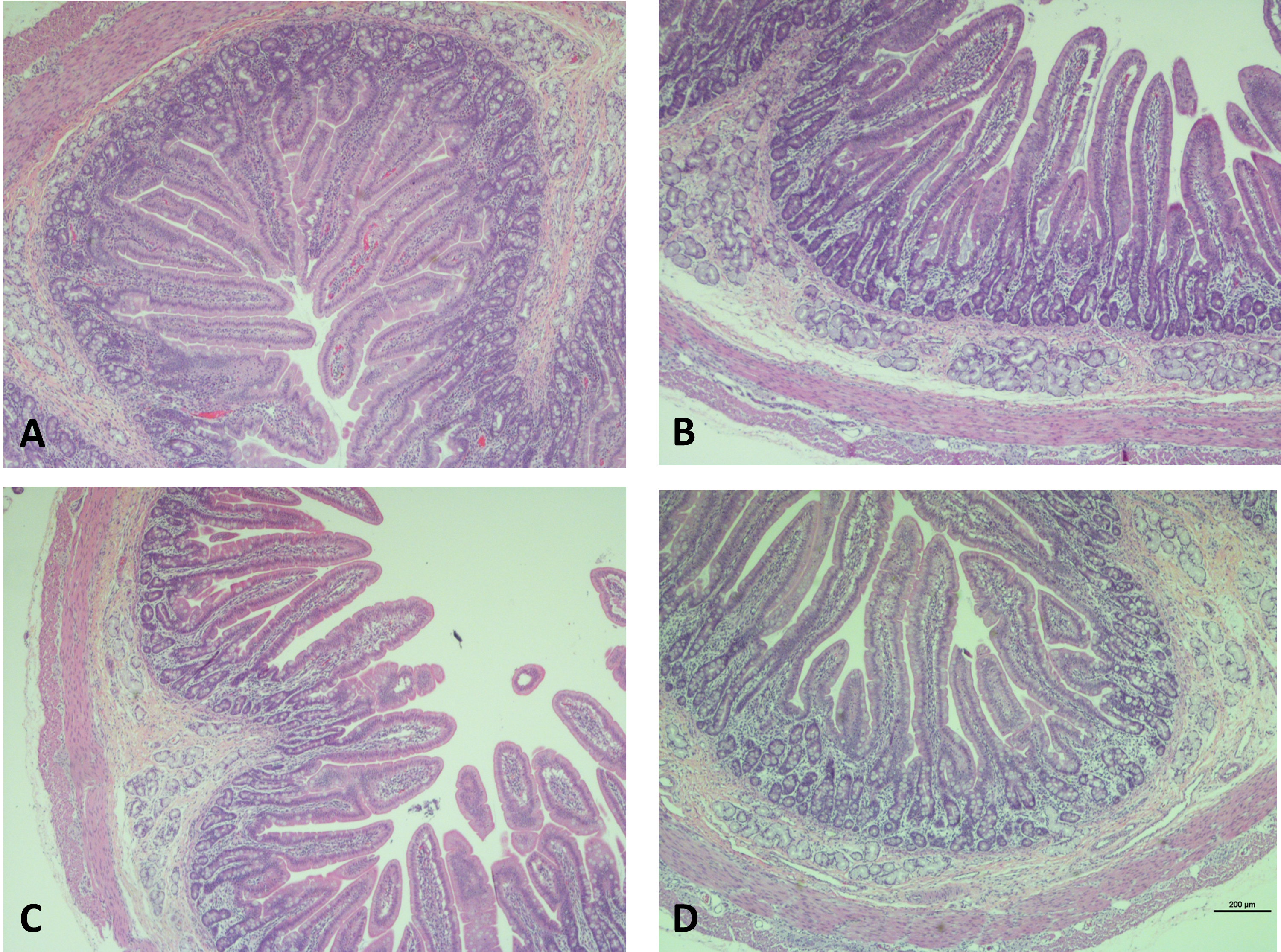

Supplement: Supplementary file 4 — Additional file 4: Figure S3. Ileum Histopathology. Representative photomicrographs of ileum sections from a pig in each group at each time point. There are no significant histologic differences between the groups. A. HHGM PID 28, B. HHGM PCD 7, C. UHGM PID28, D. UHGM PCD7. Scale bar is 200 μm. [file 13099_2016_136_MOESM4_ESM.tiff]
